# Adopting Evaluative Conditioning to Improve Coach–Athlete Relationships

**DOI:** 10.3389/fpsyg.2021.751990

**Published:** 2021-11-24

**Authors:** Jie Li, Beibei Chen, Yu Zhang

**Affiliations:** ^1^Center for Cognition and Brain Disorders, The Affiliated Hospital, Hangzhou Normal University, Hangzhou, China; ^2^Institutes of Psychological Sciences, Hangzhou Normal University, Hangzhou, China; ^3^Zhejiang Key Laboratory for Research in Assessment of Cognitive Impairments, Hangzhou Normal University, Hangzhou, China; ^4^School of Psychology, Beijing Sport University, Beijing, China; ^5^Tianjin Normal University, Tianjin, China

**Keywords:** coach–athlete relationship, evaluative conditioning, affective association, emotion, intervention

## Abstract

Coach–athlete relationships are key to athletes’ well-being, development, training, and sports performance. The present study explored the effect of an evaluative conditioning (EC) intervention on the improvement of coach–athlete relationships. We applied a 6-week EC intervention to the athletes in a volleyball team with two of their coaches involved in the EC while the third coach taken as control. In the EC, we repeatedly presented the coaches’ facial images (i.e., conditioned stimuli) together with positively valenced pictures and words (i.e., unconditioned stimuli) to the athletes. The results showed that the EC intervention led the athletes to recognize their coaches’ neutral faces as showing more happiness, respond faster to coach-positive associations in the implicit association test (IAT), and give higher ratings to the coaches in the Coach–Athlete Relationship Questionnaire (CART-Q). The present study suggests that EC may be adopted as an effective intervention for coach–athlete relationships, altering athletes’ affective associations with their coaches to be more positive and improving their explicitly evaluation of the relationship.

## Introduction

In sports, the quality of the coach-athlete relationship is of profound importance. It plays a central role in athletes’ psychosocial development and exerts major impacts upon their training, sporting performance, happiness, and welfare (Jones, 2007; Jowett and Shanmugam, 2016; Nicholls et al., 2017). To young athletes, coaches are important non-parental sources of influence, and coach–athlete relationships are key for their development in technical and physical competencies as well as psychosocial capabilities (Riley and Smith, 2011). Therefore, good coach–athlete relationships carry crucial implications of maximizing sporting and psychological outcomes in sports teams.

A coach–athlete relationship is an interconnection of emotions, thoughts and behaviors between coaches and athletes (Jowett and Ntoumanis, 2004). The 3Cs model and its extension 3+1Cs model propose that the coach–athlete relationship includes four constructs: Closeness (emotions), Commitment (thoughts), Complementarity (behaviors), and Co-orientation. Closeness refers to the emotional tone of the relationship, which encapsulates an affective bond through coaches’ and athletes’ expressions of mutual respect, trust, appreciation, and liking for one another. Commitment refers to coaches’ and athletes’ thoughts about developing lasting partnerships over time despite “ups and downs.” Complementarity refers to coaches’ and athletes’ behaviors that are complementary, cooperative, and reciprocal. Co-orientation refers to coaches’ and athletes’ interpersonal perceptions regarding the quality of the relationship (Jowett and Shanmugam, 2016).

Currently, interventions for fostering coach–athlete relationships mostly focus on behaviors, educating coaches with interpersonal skills to improve their behaviors toward athletes. For instance, the interpersonal Coach Development Programs (CDPs) have been developed, which offer coaches various learning activities applied systematically through education, social interaction and/or personal reflection with the goal of changing the coaches’ interpersonal behaviors. Sixteen categories of behavior change techniques have been developed, such as feedback and monitoring (e.g., self-monitoring of behavior), social support (e.g., practical social support), regulation (e.g., reduce negative emotions), etc. (Allan et al., 2018). For instance, researchers video recorded coaches’ reactions in games (e.g., screaming and displaying an angry facial expression to an athlete when he/she missed a shot to the goal), and then showed the videos to the coach to confront him/her with the behaviors, and meanwhile informed the coach about the differences in the athletes’ and the coaches’ answers in the on the Coach Behavior Assessment Scales. Hence, the coach could become aware of his/her behaviors and make desirable modifications accordingly (Meeûs et al., 2010). Studies have demonstrated that several interpersonal CDP trials can indeed change coaches’ behaviors and produce positive outcomes for coaches and athletes (Evans et al., 2015; Allan et al., 2018).

However, up to date, there has been little research and intervention addressing emotions in coach–athlete relationships. Emotions in interpersonal relationships sometimes feel elusive. Even if coaches and athletes deliberately endeavor to build positive emotions in their relationships, their “gut feelings” may still not feel right. This is probably because emotions contain implicit processes that occur automatically without awareness (Celeghin et al., 2020). According to the interdependence theory of interpersonal relationships, people track their positive and negative experiences with others in interpersonal relationships and weigh those experiences to form their evaluations of the relationship (Kelly and Thibaut, 1978). Furthermore, as suggested by dual-process models of social cognition, a great part of the tracking is automatic (Gawronski and Bodenhausen, 2006; Hicks and McNulty, 2019). Thus, people may automatically associate the affect derived from the positive or negative experiences with the other person in the relationship, forming affective associations (Kiviniemi et al., 2007).

Earlier research has mostly investigated people’s affective associations with objects or activities, showing that the affective associations can lead people to approach or avoid certain objects or activities promptly and automatically without deliberate thinking. For instance, studies showed that the positive or negative affect people associated with certain food, drugs, or exercises predicts whether they will consume the food, use the drugs, or engage in the exercises (Kiviniemi et al., 2007; Kiviniemi, 2018). Recent research revealed that in interpersonal relationships, affective associations are essential as well. The positive or negative affect people associated with another person is automatically activated upon meeting with or thinking about the person, and thus impact people’s judgments and behaviors in the interpersonal interactions. The affective associations are predictive of future development of the relationships, sometimes even better than deliberative self-reports (McNulty et al., 2013; McNulty and Olson, 2015). In coach–athlete relationships, athletes may also form positive or negative affective associations with coaches, which then profoundly impact the athletes’ training, sports performance, welfare, and satisfaction with the relationship (Jowett, 2017; Davis et al., 2019).

Affective associations can be altered by evaluative conditioning (EC; McNulty et al., 2017). EC refers to repeatedly pairing a stimulus (i.e., conditioned stimulus, CS) with a positive or negative stimulus (i.e., unconditioned stimulus, US), so as to associate the US’s affect with the CS. The procedure of EC is similar to Pavlovian conditioning. The difference is that Pavlovian conditioning targets changing the predictive value of the CS, whereas EC modifies the associations with the CS (Hofmann et al., 2010). In other words, EC automatically modifies people’s liking of an object due to its mere co-occurrence with other valenced objects.

While earlier research mostly investigated the effect of EC on modifying affective associations with certain foods, brand names, and various everyday objects (Hofmann et al., 2010), more recent research suggests that EC may be used to modify associations with people as well and thus benefit interpersonal relationships (Murray et al., 2011). For instance, McNulty et al. (2017) applied EC to married couples. The couples were asked to view a stream of images and words on a computer screen once every 3days for 6weeks. For the spouses in the experimental group, embedded in the stream were pictures of their partner paired with positive images (e.g., a puppy, a sunset) or words (e.g., “wonderful” and “fabulous”). The spouses in the control group viewed the same stream of images and words except that pictures of their partners were paired with neutral images or words. Results showed that the spouses who viewed their partners paired with positive stimuli demonstrated more positive results in implicit and explicit measures of the marital relationship than did control spouses.

Affective associations and explicit evaluations reflect two types of distinct yet interacting processes – associative processes and propositional processes (Gawronski and Bodenhausen, 2006). The associative processes are automatic affective reactions resulting from the particular associations that are activated automatically when one encounters a stimulus, whereas the propositional processes are syllogistic inferences derived from the propositional information that is considered relevant for a judgment. The two types of processes, respectively, build the basis for what many researchers called implicit attitude and explicit attitude. The associative processes are usually measured by spontaneous responses in implicit tasks (e.g., priming tasks, implicit association tests), whereas the propositional processes are usually measured by explicit evaluations in which participants explicitly report their agreement or disagreement with an evaluative statement about an object.

EC can exert impact on both the associative processes and the propositional processes, as suggested by the affective-propositional evaluation (APE) model, which is a representative dual-process mode (Gawronski and Bodenhausen, 2018). Repeated pairings of a CS and a US in EC produce associative links that influence spontaneous responses resulting from the spread of activation between associated concepts, leading to a transfer of affect from the US to the CS (Walther et al., 2005). The associations can be automatically activated and taken into account when making explicit evaluative judgments. Additionally, the observed co-occurrences between the CS and the US can also lead to propositional inferences about evaluative characteristics of the CS. Thus, EC effects may contain modifications in both the internal affective valence associated with the CS and the semantic memory involving the CS (Gawronski and Bodenhausen, 2018; Weber et al., 2020). Therefore, in the present study, we examined the effects of EC on both the athletes’ affective associations with their coaches and the athletes’ explicit evaluations on their relationships with the coaches.

In addition, automatic affective processes are related to the activity of the automatic nervous system (Kreibig, 2010; Weil et al., 2019). In interpersonal relationships, subordinates may perceive their superordinates as social threats. The subordinates’ reaction is faster to higher social threats compared with lower social threats, which is mediated by physiological responses of the autonomic nervous system (Behnke et al., 2020). Human’s autonomic nervous system (ANS) has evolved to support survival and social engagement. The ANS consists of the sympathetic nervous system (SNS) that is associated with physiological activation (i.e., increased arousal or “fight or flight”) and the parasympathetic nervous system (PNS) that is associated with restoration and repair (i.e., decreased arousal or “rest and digest”). The system strives to achieve a balanced state between the SNS and the PNS to optimally respond to the current environment. When a threat is perceived, the SNS is active with a suite of fight or flight responses to promotes survival. Conversely, when surroundings are deemed safe, the PNS is more active, promoting social behavior and homeostatic functions (Quintana et al., 2012). Thus, the psychosocial processes are associated with the state of the ANS, which can be indexed by various physiological measures (Cacioppo et al., 2007; Calvo and D'Mello, 2010; Yetton et al., 2019). For instance, galvanic skin response (GSR) is a measure of eccrine sweat glands innervated by the SNS, and thus increased GSR indicates increased SNS activity. Heart rate variability (HRV) measures the beat-to-beat temporal changes in the heart rate, which is an emergent property of the SNS and PNS interactive regulation. A high variability provides the flexibility to rapidly cope with the environment (Palumbo et al., 2017; Shaffer and Ginsberg, 2017). Recent research suggests that EC may be useful in modifying people’s physiological responses related to social interaction (Pawling et al., 2017). In the present study, we explored whether EC can modify athletes’ physiological responses of automatic nervous system when seeing their coaches’ faces, leading to a decrease in GSR and an increase in HRV.

In sum, in the present study, we aimed to investigate whether EC can be adopted to modify athletes’ affective associations with their coaches, explicit evaluation of their relationships with the coaches, and physiological responses when seeing the coaches, so as to improve the coach–athlete relationships. The EC intervention is complementary to prior interventions targeting coaching behaviors in coach–athlete relationships. Rather than changing coaches’ behaviors, this approach is about changing athletes’ perceptions of their coaches based on automatic processes. In current research on interpersonal relationships, there is still a lack of interventions on the automatic processes (Faure et al., 2020; Van Dessel et al., 2020). Whereas EC interventions have been shown to be effective in marital relationships, its effectiveness in other areas of interpersonal relationships has not been examined. Different from marital relationships, coach–athlete relationships work in group circumstances. Various factors at the group level may influence the relationships between the coaches and athletes, such as group dynamics, training schedules, and team performance in recent competitions, masking the effects of interventions at the individual level. Thus, in the present study, we conducted the intervention on the athletes in one sport team with some of their coaches involved in the EC intervention with the other coaches taken as control; additionally, we explored the effect of EC on coach–athlete relationships by testing multiple measures *via* varied procedures, including emotion recognition, IAT, questionnaires, face judgments, and physiological measurement.

## Materials and Methods

### Participants

A junior female volleyball team was tested. There were 19 athletes in the team, along with 3 coaches. The athletes’ average age was 15.63 (*SD*=1.32), and their average year of training was 3.12 (*SD*=1.02). The coaches were all males (age range 25–38years). Two athletes quit the team during the study, so that there were complete data of 17 athletes. The athletes and coaches provided informed consent prior to participation. The institutional ethics committee approved the study. The results were kept confidential, only used for the research. The experimental procedure was conducted individually to each athlete by the experimenter (i.e., the second author who worked in the team as a sport psychologist at the time) in a quiet room in the training center of the team. The athletes were informed that if feeling uncomfortable they could skip a test or quit the procedure at any time and their data would not be revealed to the coaches or anyone else outside the research group. The athletes were fully debriefed about the study after the intervention and approved their data to be analyzed and published.

### Experimental Procedure and Design

**Figure 1:**
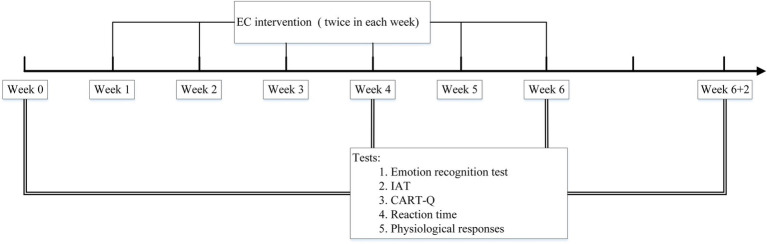
The timeline of the EC intervention and the tests. The EC intervention was conducted twice a week for 6weeks (Week 1–6). The measures were tested before the intervention (Week 0), during the intervention (Week 4), at the end of the intervention (Week 6), and post intervention (Week 6+2).

We performed EC interventions on the athletes twice a week for 6weeks. Each athlete received EC interventions for two randomly selected coaches and no intervention for the other coach. In this way, the same athletes’ relationships with the EC-intervened and non-intervened coaches were compared, so that we could minimize possible influence of other factors on the coach–athlete relationships. Before (Week 0), during (Week 4), and at the end of the EC (Week 6), as well as post-EC (Week 6+2, i.e., 2weeks after the EC ended), the following measures were tested on the athletes (see Figure 1). We measured the athletes’ affective associations with the coaches by using biases in an emotion recognition test (Maner et al., 2005; Krems et al., 2015) and an implicit association test (IAT; Greenwald et al., 2003; Karpinski and Steinman, 2006). We measured the athletes’ explicit evaluation of their relationships with the coaches by using the Coach–Athlete Relationship Questionnaire (CART-Q; Jowett and Ntoumanis, 2004; Zhong and Wang, 2010), in which the athletes explicitly give ratings to a series of evaluative statements about the relationships. We also tested the athletes’ reaction time and physiological responses of automatic nervous system (indexed by GSR and HRV; Quintana et al., 2012) when viewing the coaches’ facial images in a face orientation judgment task.

### Experimental Tasks, Materials, and Apparatus

#### The EC Intervention

The selection of the stimuli and the procedure of the EC followed McNulty et al.'s (2017) protocol. The stimuli included CSs, USs, target stimuli, neutral irrelevant stimuli, along with blank screens interleaved in the stream. The CSs were the coaches’ neutral facial images from front view. The USs were positive pictures depicting endearing animals, beautiful natural scenes, or delicious desserts, and positive words describing good personal characteristics. The target stimuli were photos showing coaches and athletes together in a good relationship and words describing good relationships. The neutral irrelevant stimuli were pictures of neutral objects from the UCSD Vision and Memory Lab database (Brady et al., 2008) and names of daily household objects. For the selection of the stimuli, we firstly gathered 55 words and 55 pictures of each type of stimuli as a pool. Then, we had the stimuli rated on a 7-point scale (1-very negative, 4-neutral, and 7-very positive) by 57 students who did not participate in the experiment. Based on the rating, 36 words and 39 pictures of each type were selected. The mean ratings of the selected US pictures and US words were both 5.8 (*SD*=0.2 and 0.1, respectively), while the mean ratings of the neutral words and neutral pictures were both 4.1 (*SD*s=0.2).

EC interventions were conducted on the athletes individually twice a week for 6weeks. Each athlete received EC interventions for two randomly selected coaches and no intervention for the other coach. The randomization was conducted for each athlete so that different athletes could receive interventions for different coaches. In the EC, the athletes were presented streams of pictures and words. Each EC intervention session contained 5 blocks for each of the two selected coaches, lasting for about 15min in total. In each block, the CS (i.e., the coach’s face) was presented simultaneously with a US (i.e., a positive picture or word) side by side for 5 times, preceded and followed by blank screens. The target stimuli were presented alone 3 times and presented simultaneously with a neutral irrelevant stimulus twice. The neutral irrelevant stimuli were presented alone 10 times. The blank screen was presented alone 5 times. Each stimulus was presented for 1,500ms. The order of the stimuli was random. The athletes were required to press “j” on the keyboard when seeing the target stimuli, which was actually a cover task for having the athletes to pay attention to the stream of stimuli.

#### The Emotion Recognition Task for Testing Affective Associations

We tested the athletes’ affective associations with the coaches by examining their biases in recognizing emotional expressions on the coaches’ neutral faces (Maner et al., 2005; Krems et al., 2015). In the test, the three coaches’ neutral front faces were displayed on screen one by one with the order randomized for each athlete. The athletes were required to indicate the extent to which they perceived each of the six basic emotions (i.e., happiness, anger, disgust, sadness, fear, surprise) on each coach’s face by using 7-point scales (1-not at all, 7-very much). In reality, the coaches wore a neutral face when taking the photo, and the faces were rated as neutral by the aforementioned 57 students who had no acquaintance with the coaches, scoring 4.02 on average on a 7-point scale (1-very negative, 7-very positive). The biases reflect each athlete’s systematic distortion in perceiving each coach’s facial expressions.

#### The IAT Task

In IAT paradigms, target concepts or attribute words are presented on a computer screen, assigned with either the same response key or different response keys. If a concept and an attribute are associated positively in the participant’s mind, the participant will respond faster when they are assigned with the same key than different keys. The difference in the reaction time between the two conditions can be taken as the measure for the direction and strength of the associations (Greenwald et al., 2003).

We adopted the Single-Category Implicit Association Test (SC-IAT), which is an adapted version of the classical IAT for measuring the association between a single target and relevant attributions (Karpinski and Steinman, 2006). The coaches’ faces served as target, while 10 positive words (e.g., supportive, respect) and 10 negative words (e.g., neglect, autocratic) related to coach–athlete relationships were selected as attributions. The positive and negative words were rated 6.4 (*SD*=0.3) and 1.7 (*SD*=0.2) on average, respectively, on a 7-point scale (1-very negative, 7-very positive) by the 57 students aforementioned.

Each athlete conducted three SC-IAT sessions separately for the three coaches in randomized orders. Each session consisted of four blocks. The athletes responded to the coach’s face by pressing the same key as to the positive words (in Blocks 1 and 2) or the negative words (in Blocks 3 and 4). Blocks 1 and 3 served as practice blocks, each containing 24 trials, while Blocks 2 and 4 were experimental blocks, each containing 72 trials. In Blocks 1 and 2, the ratio of the coach’ face, the positive words, and the negative words was 7:7:10, while in Block 3 and 4, the ratio was 7:10:7, so as to reduce possible preferences in key pressing responses (Karpinski and Steinman, 2006). Trials in which the athletes’ responses were incorrect or the reaction time was shorter than 300ms or outside 3 standard deviations of the mean were excluded from analyses. The average reaction time of Block 2 (i.e., coach associated with positive words) was subtracted from that of Block 4 (i.e., coach associated negative words) and then divided by the standard deviation of the reaction time of all the valid trials in Block 2 and 4, resulting in the *D* score of the SC-IAT task (Karpinski and Steinman, 2006). Higher *D* represents stronger positive implicit associations with the coach.

#### The CART-Q Questionnaire

The athletes’ explicit evaluation of their relationships with the coaches was measured by the Coach–Athlete Relationship Questionnaire (CART-Q), which has been widely used for assessing coach–athlete relationships in many countries around the world (e.g., United Kingdom, China, Greece; Yang and Jowett, 2012). In the present study, the Chinese version of the CART-Q questionnaires (Zhong and Wang, 2010) was used. The questionnaire consists of 20 items measuring the three dimensions of the coach–athlete relationships: emotions (6 items; e.g., Do you like your coach?), thoughts (7 items; e.g., I feel that my sport career is promising with my athlete/coach), and behaviors (7 items; e.g., When I am coached by my coach, I am ready to do my best). Each item was rated on a 7-point scale (1-Not-at-all, 7-Extremely). Higher scores indicate better coach–athlete relationships.

#### The Face Orientation Judgment Task

The face orientation judgment task was used as a paradigm for presenting coaches’ faces in repeated trials so as to obtain athletes’ stable reaction time and physiological responses upon viewing the coaches’ faces. The procedure was similar to that used in Ma and Han’s study for examining “boss effect” (Ma and Han, 2009). We took 10 photos of each coach’s neutral face rotating left or right for approximately 30, 45, 60, 75, or 90° as the stimuli. In the experiment, firstly, a central fixation point was presented for 200ms, followed by an image of a coach’s face rotated left or right or a black image with a vertical gray bar on the left or right side. The athletes were required to judge as accurately and quickly as possible whether the face rotated to left or right, or whether the bar was on the left or right side, by pressing “D” or “F” respectively on the keyboard with their left hand. The image disappeared upon the athlete’s key pressing or after 1,500ms without response, and then, the next trial started. The athletes’ reaction time in each trial was recorded, and their GSR and HRV were recorded throughout the experiment by the NEXUS-10 MARK II equipment. Two GSR sensors were attached to the athlete’ index and middle fingers on the right hand, and a heart rate sensor was attached to the athlete’s thumb on the right hand, recording the athlete’s GSR and heart beat continuously during the face orientation judgment experiment. The standard deviation of consecutive normal beats (SDNN) was analyzed, as it reflects overall HRV (Quintana et al., 2012).

There were six blocks in the experiment, three blocks for the three coaches’ faces separately, interleaved by three blocks for the black images with bars. Each face block contained 40 trials, while each bar block contained 18 trials. The bar blocks were used to test the athletes’ baseline reaction time and physiological responses in the orientation judgment task (Ma and Han, 2009). The GSR and the HRV throughout each block was calculated by using the v2011BioTrace+software as the scores for each athlete’s physiological responses to each coach’s face or the bar. By subtracting the athletes’ reaction time, GSR, and HRV when judging the direction of the bar from those when judging the orientation of the coaches’ faces, we obtained the measures reflecting the effects of the coaches’ faces.

### Data Analyses

We analyzed both the individual effects of the EC intervention on each athlete and the overall effects across the athletes. We examined the individual effect by visually inspecting the progress of each athlete’s relationship with each coach along with the EC intervention. And we computed the percentages of athletes showed improvement in each measure at the end of the EC intervention (i.e., Week 6) compared to before the intervention (i.e., Week 0). We conducted 4 (testing time: Week 0, Week 4, Week 6, Week 6+2)×2 (intervention status: EC intervention, no intervention) mixed two-way analyses of variance (ANOVAs) on each measure to analyze the overall effects of the EC intervention with time.

## Results

### Individual Progresses With the EC Intervention

**Figure 2:**
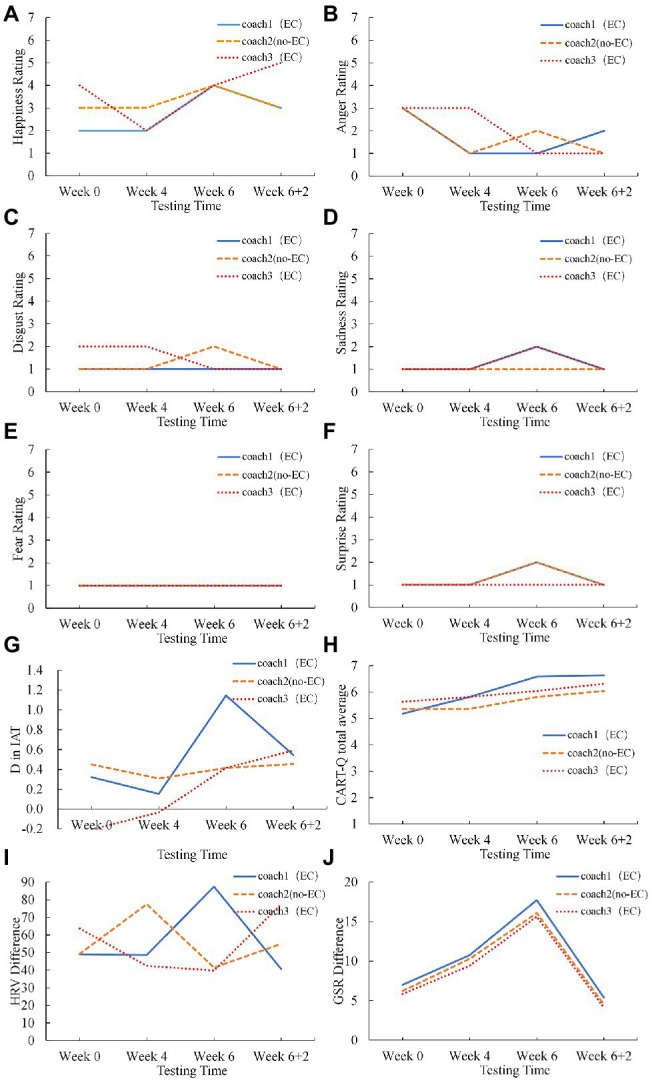
The variation of Athlete 1’s **(A–F)** the emotion ratings (happiness, anger, disgust, sadness, fear, surprise), **(G)** the D score in IAT, **(H)** the CART-Q scores, **(I)** the HRV, and **(J)** the GSR with time for the coaches with and without EC.

**Figure 3:**
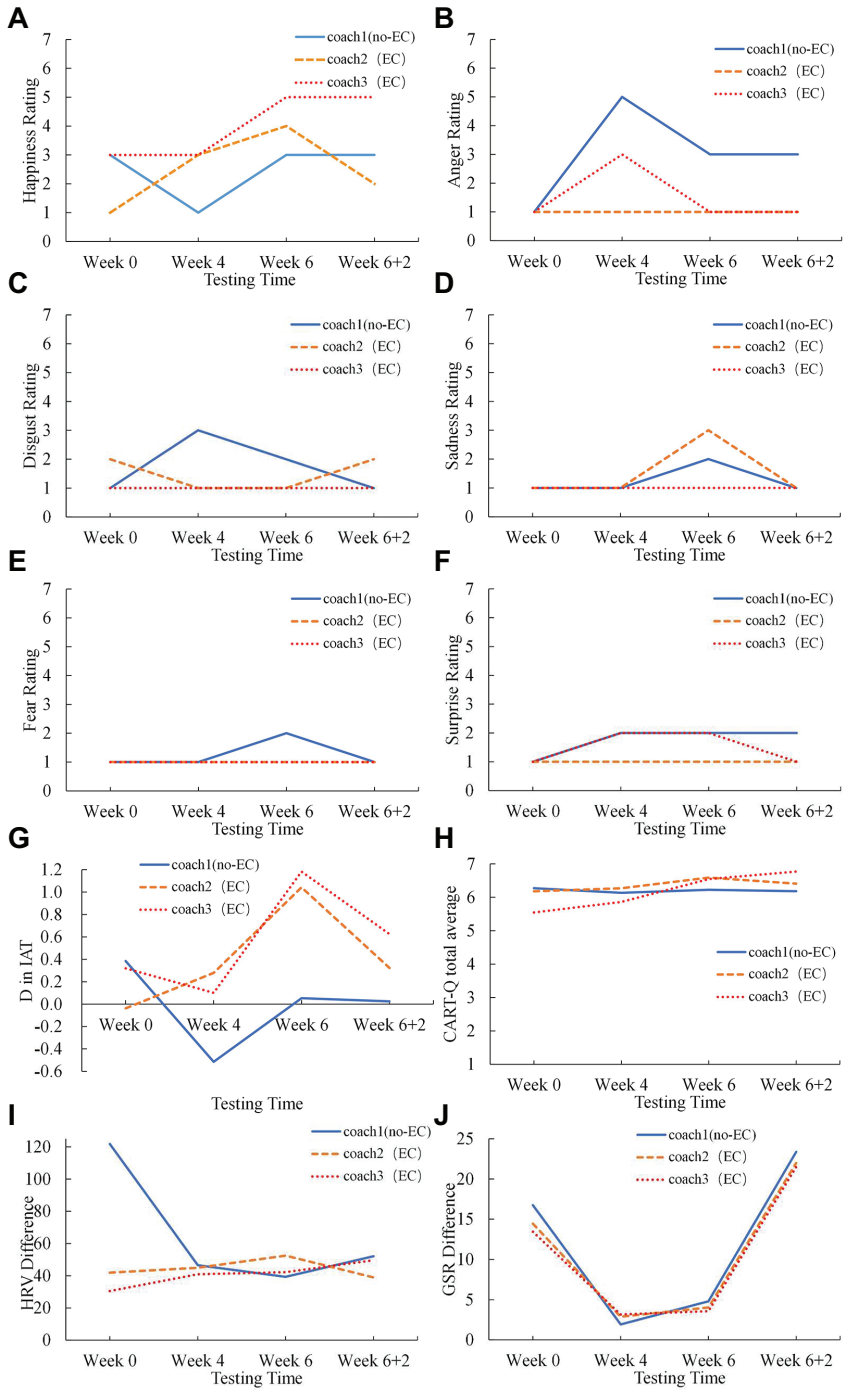
The variation of Athlete 2’s **(A–F)** the emotion ratings (happiness, anger, disgust, sadness, fear, surprise), **(G)** the D score in IAT, **(H)** the CART-Q scores, **(I)** the HRV, and **(J)** the GSR with time for the coaches with and without EC.

We visually inspected the variation of each measure regarding each athlete’s relationship with each coach along with the EC intervention. Figures 2, 3 depicted the progresses of two athletes’ relationships with the coaches with and without the EC intervention. The athletes’ progresses showed similarities and differences.

Specifically, on the emotion recognition biases, many athletes showed a relatively high rating with a large fluctuation in happiness. For example, Athlete 2 exhibited an increase in the happiness rating for the two coaches involved in EC, while there was no increase (and even a temporary decrease at Week 4) in the happiness rating for the coach that was not involved in EC (Figure 3A). Complementary to the changes in the happiness rating, Athlete 2 exhibited a remarkable increase in the anger rating for the coach without EC at Week 4, which extended to Week 6 and Week 6+2, while the anger rating for one EC coach remained at the lowest level and the anger rating for the other EC coach temporarily increased at Week 4 yet then decreased to the lowest level (Figure 3B). Athlete 1 exhibited continuous increase in the happiness rating for one EC coach while the happiness rating for the other two coaches fluctuated in an increasing trend (Figure 2A); meanwhile, the anger ratings for the three coaches fluctuated in a decreasing trend (Figure 2B). On the ratings of disgust, sadness, fear, and surprise, most athletes scored at low levels with small variation (see Figures 2C–F, 3C–F as examples).

On the IAT score, most athletes exhibited increases for the EC coach and no changes for the no-EC coach (e.g., Athlete 1 as depicted in Figure 2G). Additionally, Athlete 2’s IAT score on the no-EC coach exhibited a decrease at Week 4 (Figure 3G), consistent with the athlete’s decreased happiness rating and increased anger rating with the no-EC coach at Week 4.

On the CART-Q questionnaire, most athletes rated high scores with all the three coaches. Athlete 1 exhibited a small but steady increasing trend in the CART-Q for all the three coaches (Figure 2H). Athlete 2 exhibited an increasing trend in the CART-Q for the EC coaches, while the score remained unchanged for the no-EC coach (Figure 3H). Interestingly, Athlete 2’s explicit evaluation of the relationship with the no-EC coach (i.e., CART-Q score) remained unchanged at a high level even at Week 4, while the implicit measures of the relationship (i.e., happiness rating, anger rating, IAT) exhibited a substantial decrease.

Athletes’ physiological responses upon seeing the coaches’ faces exhibited no clear patterns. For example, Athlete 1’s HRV with all the three coaches fluctuated (Figure 2I), whereas Athlete 2’s HRV remained largely stable except a sharp decrease from Week 0 to Week 4 for the no-EC coach (Figure 3I). Athlete 1’s GSR for all the three coaches increased from Week 0 to Week 6 and then decreased at Week 6+2 (Figure 2J), while Athlete 2’s GSR for all the three coaches decreased at Week 4 and Week 6 and then increased at Week 6+2 (Figure 3I).

### Frequency Analyses

We calculated the percentages of athletes that showed increases at Week 6 (i.e., at the end of EC) in comparison with Week 0 (i.e., before EC) for the coaches with or without the EC intervention in each measurement (see Table 1). The results showed the trends that EC led to improvements in a considerable portion of athletes on the happiness rating, the IAT, and the CART-Q measurements. In contrast, a large portion of athletes showed increases on the anger and the disgust ratings for the coaches without EC, while only a few athletes showed such increases for the coaches with EC.

**Table 1:** The percentages of athletes that showed increases at Week 6 (i.e., at the end of EC) in comparison to Week 0 (i.e., before EC) for the coaches with or without the EC intervention in each measurement, including the emotion recognition biases, the Implicit Association Test (IAT), the Coach–Athlete Relationship Questionnaire (CART-Q), and the galvanic skin response (GSR) and SDNN measure of the heart rate variability (HRV) when viewing the coaches’ faces.

Measurement	No EC (%)	EC (%)
Emotion recognition biases		
*Happiness*	17.6	61.8
*Anger*	82.4	20.6
*Disgust*	70.6	8.8
*Sadness*	35.3	38.2
*Fear*	11.8	23.5
*Surprise*	29.4	32.4
IAT	35.3	91.2
CART-Q	35.3	58.8
GSR	5.9	14.7
HRV	52.9	44.1

### The Overall Effects of EC on Emotion Recognition Biases

We examined the overall effects of EC on the athletes’ biases in recognizing coaches’ emotions by conducting a set of 4 (testing time: Week 0, Week 4, Week 6, Week 6+2)×2 (intervention status: EC intervention, no intervention) mixed two-way ANOVAs separately on the ratings of happiness, anger, disgust, sadness, fear, and surprise, with the testing time as the within-participants variable and the intervention status as the between-participants variables. The results are listed in Table 2.

**Table 2:** The Analysis of Variance Statistics for the Emotion Recognition Test.

Emotion	Variables	*df*	*F*	*p*	η_p_^2^
Happiness	Testing Time	3	0.687	0.561	0.014
	Intervention Status	1	1.527	0.222	0.030
	Testing Time×Intervention Status	3	3.579	0.015	0.068
Anger	Testing Time	3	3.619	0.015	0.069
	Intervention Status	1	9.031	0.004	0.156
	Testing Time×Intervention Status	3	5.446	0.001	0.100
Disgust	Testing Time	3	5.411	0.003	0.099
	Intervention Status	1	8.274	0.006	0.144
	Testing Time×Intervention Status	3	6.735	0.001	0.121
Sadness	Testing Time	3	1.555	0.220	0.031
	Intervention Status	1	0.020	0.888	0.000
	Testing Time×Intervention Status	3	0.330	0.679	0.007
Fear	Testing Time	3	0.574	0.577	0.012
	Intervention Status	1	2.180	0.146	0.043
	Testing Time×Intervention Status	3	0.857	0.434	0.017
Surprise	Testing Time	3	2.066	0.120	0.040
	Intervention Status	1	0.213	0.647	0.004
	Testing Time×Intervention Status	3	0.092	0.944	0.002

**Figure 4:**
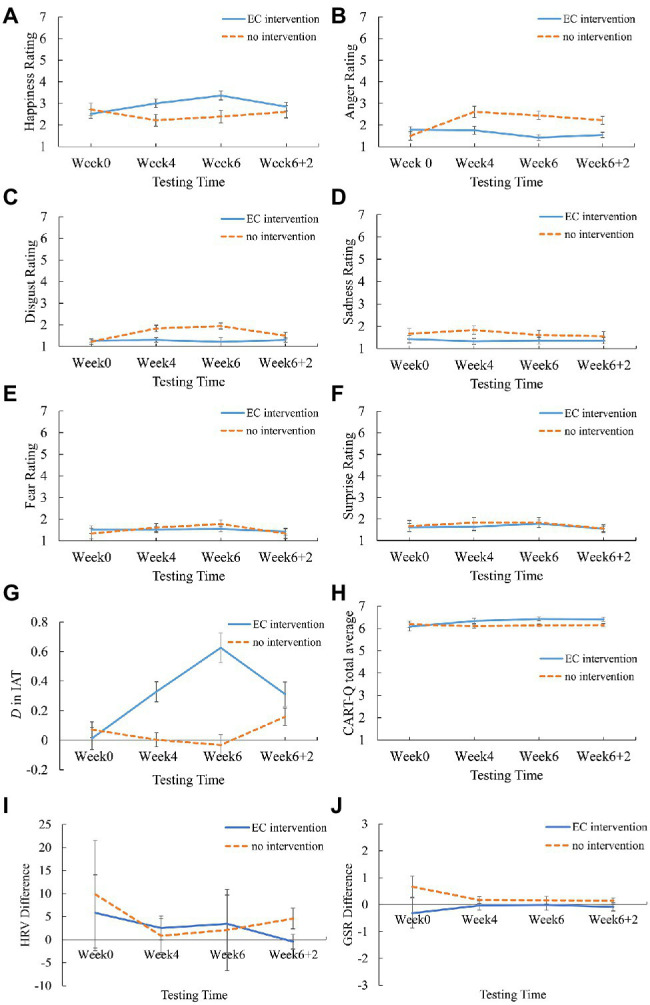
The variation of **(A–F)** the emotion ratings (happiness, anger, disgust, sadness, fear, surprise), **(G)** the D score in IAT, **(H)** the CART-Q scores, **(I)** the HRV, and **(J)** the GSR with time in the EC intervention and no intervention conditions.

The key results were that the Testing Time × Intervention Status interactions were significant for happiness, anger, and disgust ratings (*p*s<0.015; see Figure 4). Further analysis showed that for the EC-intervened coaches, the main effect of testing time on happiness was significant, *F*(3, 135)=3.577, *p*=0.021, η_p_^2^=0.186, showing a higher happiness rating at Week 4 than Week 0 (3.32 vs. 2.50, *p*=0.012) while no significant difference in the comparisons between the other weeks (*p*s>0.141). For the non-intervened coaches, the main effect of testing time on happiness was not significant. On the other hand, for the non-intervened coaches, the main effect of testing time on anger [*F*(3, 135)=5.589, *p*=0.002, η_p_^2^=0.263] and disgust [*F*(3, 135)=10.480, *p*<0.001, η_p_^2^=0.401], both showing higher ratings at Week 4, 6, and 6+2 in comparison with Week 0 (2.61, 2.52, 2.22 vs. 1.57, *p*=0.005, 0.008, 0.025; 1.83, 2.00, 1.47 vs. 1.24, *p*=0.002, *p*<0.001, *p*=0.033, respectively for anger and disgust). None of the other comparisons were significant (*p*s>0.313). For the EC-intervened coaches, the main effects of testing time on anger and disgust were not significant. On the sadness, fear, and surprising ratings, the ANOVAs did not yield any significant main effects or interactions (*p*s>0.120). The results demonstrated that for the coaches with EC intervention, the happiness association increased with time while the anger and disgust associations remained unchanged, whereas for the coaches without EC intervention, the anger and disgust associations deteriorated with time while the happiness association remained unchanged (see Figure 4).

### The Overall Effects of EC on IAT

Analogous to the analyses above, we conducted a 4 (testing time)×2 (intervention status) mixed two-way ANOVA on the D score in the SC-IAT task. The main effects of testing time and intervention status were significant, and more importantly, the Testing Time × Intervention Status interaction was significant (*p*<0.001, see Table 3). Further analysis showed that for the EC-intervened coaches, the Ds at Week 4, 6, and 6+2 were all significantly higher than that at Week 0 (0.32, 0.62, 0.30 vs. 0.04, *p*<0.001, *p*<0.001, *p*=0.003, respectively), and the D at Week 6 was significantly higher than that at Week 4 (0.62 vs. 0.32, *p*=0.001) and that at Week 6+2 (0.62 vs. 0.30, *p*=0.001), while there was no significant difference between Week 4 and Week 6+2. For the non-intervened coaches the D score did not vary with time, *F*(3, 135)=1.940, *p*=0.136, η_p_^2^=0.110. The results demonstrated that the athletes’ implicit associations with the coaches become more and more positive as the implementation of the EC intervention, and then descended after the intervention stopped (see Figure 4).

**Table 3:** The Analysis of Variance Statistics for the Implicit Association Test (IAT), the Coach–Athlete Relationship Questionnaire (CART-Q), and the Athletes’ Reaction Time, Galvanic Skin Response (GSR) and SDNN Measure of the Heart Rate Variability (HRV) When Viewing the Coaches’ Faces.

Measurement	Variables	*df*	*F*	*p*	η_p_^2^
IAT	Testing Time	3	5.014	0.002	0.093
	Intervention Status	1	18.253	0.000	0.271
	Testing Time×Intervention Status	3	13.004	0.000	0.210
CART-Q	Testing Time	3	1.648	0.181	0.033
	Intervention Status	1	1.827	0.183	0.036
	Testing Time×Intervention Status	3	2.893	0.037	0.056
Reaction Time	Testing Time	3	3.908	0.010	0.074
	Intervention Status	1	0.176	0.677	0.004
	Testing Time×Intervention Status	3	0.393	0.758	0.008
GSR	Testing Time	3	0.189	0.736	0.004
	Intervention Status	1	2.370	0.130	0.046
	Testing Time×Intervention Status	3	1.495	0.231	0.030
HRV	Testing Time	3	0.459	0.642	0.009
	Intervention Status	1	0.102	0.750	0.002
	Testing Time×Intervention Status	3	0.201	0.828	0.004

### The Overall Effects of EC on Explicit Evaluation of Coach–Athlete Relationships

Analogous to the analyses above, we conducted a 4 (testing time)×2 (intervention status) mixed two-way ANOVA on the average score of CART-Q. The results showed that the Testing Time × Intervention Status interaction was significant (*p*=0.037), while the main effects were not significant (*p*s>0.181; see Table 3). Further analysis showed that for the EC-intervened coaches, the CART-Q scores at Week 6 and Week 6+2 were significantly higher than that at Week 0 (6.44, 6.41 vs. 6.09, *p*=0.025, 0.020, respectively), while there was no significant difference between Week 4, 6, and 6+2 (*p*s>0.075). For the non-intervened coaches, the CART-Q score did not vary with time, *F*(3, 48)=0.263, *p*=0.852, η_p_^2^=0.016. The results demonstrated that the EC intervention improves the athletes’ explicit evaluation of their relationships with the coaches (see Figure 4). We also performed repeated measure ANOVAs on the three dimensions of the CART-Q (i.e., emotion, thoughts, and behaviors) separately. The result patterns of the three dimensions were all similar to that of the total score, showing trends of improvement for the EC coaches and no improvement for the no-EC coach. Yet none of the effects reached significant (*p*s>0.111) in the dimensions of thoughts and behaviors. In the emotion dimension, the main effect of testing time was significant, *F*(3, 147)=3.958, *p*=0.009, η_p_^2^=0.075, and the Testing Time × Intervention Status interaction approached significant, *F*(3, 147)=2.378, *p*=0.072, η_p_^2^=0.046, suggesting that the emotion dimension may be relatively sensitive to the EC intervention.

### The Overall Effects of EC on Athletes’ Reaction Time and Physiological Responses When Viewing Coaches’ Faces

We firstly subtracted the athletes’ reaction time, GSR, and HRV when judging the direction of the bar from those when judging the orientation of the coaches’ faces, so as to obtain the measures reflecting the effects of the coaches’ faces. Then, analogous to the analyses above, we conducted 4 (testing time)×2 (intervention status) mixed two-way ANOVAs on these measures. The Testing Time×Intervention Status was not significant for any of the measures (*p*s>0.231; Table 3), indicating that the EC intervention did not lead to significant changes in the athletes’ reaction time and physiological responses when viewing the coaches’ faces.

## Discussion

The present study showed that a six-week EC intervention to athletes improved their relationships with coaches. Specifically, the EC intervention benefited the athletes’ affective associations with the coaches by increasing the happiness association and preventing the anger and disgust associations from deterioration; meanwhile, the athletes’ implicit associations with the coaches as measured by IAT became more positive. In addition, the athletes’ explicit evaluation of their relationships with the coaches as measured by CART-Q improved as well. On the other hand, the athletes’ reaction time, GSR, and HRV when viewing the coaches’ faces were not significantly impacted by the EC intervention. These results provide novel evidence for a mechanism of change in coach–athlete relationships and suggest novel avenues for interventions. Below we discussed the implications in detail.

### Affective Associations in Coach–Athlete Relationships

The present study illustrated the importance of affective associations in coach–athlete relationships. As suggested by the interdependence theory of interpersonal relationship (Kelly and Thibaut, 1978) and the dual-process models (Smith and DeCoster, 2000; Gawronski and Bodenhausen, 2006; Hicks and McNulty, 2019), in daily interactions with coaches, athletes may automatically track their positive and negative experiences. If a coach’s presence is frequently accompanied with positive/negative events and affect, athletes may establish associations between the coach and the positive/negative affect. These affective associations are automatically activated whenever people encounter or merely think about the partner, and thus impact their judgments in the interpersonal interactions (McNulty and Olson, 2015; Hicks and McNulty, 2019). If an athlete has established a negative association with a coach, the athlete would be biased to judge that the coach is expressing anger and disgust to him/her even when the coach is in a neutral state.

Affective associations may further impact the athletes’ behaviors and thoughts toward the coaches. Affective associations are automatically activated in milliseconds without conscious awareness, and the activated affect directly signal to the individual how to behave with respect to the stimulus (Fazio, 1990). Athletes may automatically approach the coaches associated with positive affect and behave in a positive manner, whereas they may automatically start the ‘fight or flight’ response towards the coaches associated with negative affect. Moreover, the activated affect can also impact the information processing related to the stimulus and the cognitive beliefs about the stimulus (Kiviniemi et al., 2007). Specifically, the affect can bias information processing by guiding attention (Roskos-Ewoldsen and Fazio, 1992). When an athlete has formed negative associations with a coach, he/she may selectively process the coach’s negative information and neglect positive information, which will confirm and exacerbate the negative bias. His/her cognitive belief about the coach may also be attuned with the negative information to justify the negative affect, so that the athlete may consciously form the belief that the coach is a ‘bad’ person who deliberately makes him/her feel bad.

### Adopting EC Intervention to Improve Coach–Athlete Relationships

The present study suggests that EC may be adopted to modify affective associations and improve explicit evaluation of coach–athlete relationships. In the present EC procedure, a coach repeatedly appeared with objects of positive affect, which efficiently added weight to athletes’ positive associations with the coach and prevented negative associations from deterioration. Furthermore, according to the dual-process models (Kelly and Thibaut, 1978; Gawronski and Bodenhausen, 2006; Hicks and McNulty, 2019), the affective associations can be automatically activated and taken into account when athletes deliberately evaluate their relationship with the coaches. Thus, as EC modified the implicit associative structures, the athlete’s explicit evaluation showed corresponding changes (Gawronski and Bodenhausen, 2006; Zerhouni et al., 2018). It is noteworthy that whereas the intervention stemmed from the dual-process theories, the consistent changes in the implicit and explicit measures can also be explained by single-process theories (e.g., Cunningham et al., 2007; Fazio, 2007). According to the single-process theories, the construct validity of the implicit measures such as IAT is questionable (Van Dessel et al., 2020; Schimmack, 2021), as both the implicit and explicit measures may reflect largely overlapping or unitary mental content, rather than explicit measures tapping explicit constructs and implicit measures tapping implicit constructs (De Houwer, 2014; Kurdi et al., 2021). In the present study, multiple measures were adopted not to test the dual-process and single-process theories, but to extensively explore the potential effects of EC on coach–athlete relationships from different aspects. The consistent changes in the measures shed light on the growing body of research on developing novel interventions in close relationship (McNulty et al., 2017; Faure et al., 2020).

An interesting finding in the present study is that the anger and disgust associations increased over time for the coaches without EC intervention while remained unchanged for the EC–intervened coaches. The result implies that the coach–athlete relationships in sports teams may tend to decline over time if not managed with extra care. Similar findings have been reported in research on marital relationships, showing that many married people evaluate their relationships less positively over time. The decline persists even in spouses with motivations to perceive their relationships positively and cognitive strategies that appear to help them do so (Hicks and McNulty, 2019). So far, research on the development of coach–athlete relationships over time is limited. Consistent with the marital relationships research, a case study discovered a regressive spiral in coach–athlete relationships after an initial ‘honeymoon’ phase (Jowett, 2003). In marital relationships, spouses with more positive associative processes are less likely to experience the decline (McNulty et al., 2013). In coach–athlete relationships, this may also be the case. Therefore, an EC intervention may be a helpful and needful tool for maintaining positive coach–athlete relationships over time. On the other hand, it should also be noted that coach–athlete relationships differ from marital relationships in a number of ways. For instance, a coach–athlete relationship is always one of uneven power and it usually occur in a group situation. Thus, it is better for interventions to be adapted accordingly. Additionally, the present results exhibited a decreasing trend in several measures on coach–athlete relationships after the EC intervention ceased, whereas previous research suggested that the effects of EC are resistant to extinction when strong CS–US contingency is no longer presented (Díaz et al., 2005; Bolders et al., 2012; Hütter et al., 2012). There has been a controversy on the extinction of EC effects (Hofmann et al., 2010; Gawronski et al., 2015). The present trend implied that some daily experiences with coaches may add weight to the negative associations in the coach–athlete relationships. Therefore, in coach–athlete relationships, the effect of EC interventions (and other interventions as well) may need to be reinforced from time to time.

Using EC to improve coach–athlete relationships in sports teams has its distinct advantages. Firstly, EC can impact the automatic processes that cannot be addressed by traditional interventions that address explicit behaviors only. EC strengthens positive affective associations in memory, which can further lead the individual to automatically perceive the stimulus in a more positive manner in future encounters (Gawronski and Bodenhausen, 2018). Secondly, EC does not cost much cognitive resource (Hütter et al., 2012). The EC intervention requires mere co-occurrence of the target person with valenced objects or events. In the present EC procedure, athletes were only conducting a simple search task while the EC produces effects incidentally through repeated paring of the coaches’ faces and positive stimuli.

### Limitations and Future Directions

In the present study, the EC intervention did not lead to significant changes in the athletes’ reaction time and physiological responses when viewing their coaches’ faces. It is possible that EC does not reduce perceived social threat from the coaches’ faces or longer EC interventions are needed for altering the reaction time and physiological responses. Additionally, the present study suggests that there may be limitations in adopting physiological measures to examine coach–athlete relationships, as the data may be noisy and prone to interferences from movement, sweat, or electromagnetic sources (Palumbo et al., 2017; Yetton et al., 2019). Moreover, the physiological data may not directly map to the people mental states, and different people may have different patterns of physiological responding (Vasilev et al., 2009; Calvo and D'Mello, 2010). Therefore, researchers in future studies may need to develop more delicate methods to collect and analyze the physiological data, and interpret the results cautiously.

Additionally, in order to control possible confounding factors regarding coach–athlete relationships, we tested athletes from one team and randomly assigned their multiple coaches to either the EC intervention condition or no intervention condition. This resulted in a limited number of participants in the study. The reliance upon a single team of athletes and a small number of target coaches limited the robustness and generalizability of the study. Thus, the present study is still exploratory. The interpretation of and the inferences from the results should be made cautiously. The present study can serve as a starting point for further intervention on larger scale. Future studies may extend this line of research by testing more athletes in more sports teams.

On the other hand, it should also be noted that in real life, each athlete is a unique individual, and so is each coach. Each coach–athlete relationship may evolve in a unique way based on various factors, such as the athlete’s skills and competitive level, the coach’s behavior style and authority, and the athlete’s and the coach’s personalities and experiences. Thus, in sport psychology practices, it is impractical to abstract the coach–athlete relationship and adopt a one-size-fit-all method to conduct interventions on all relationships. The present study suggests that EC may be effective for a considerable portion of athletes, and hence it could be a potential tool for sport psychologists to adopt when dealing with certain coach–athlete relationships. Nevertheless, individual effect needs to be stressed and individualized interventions are better to be developed for each relationship.

Future study may explore the ways to incorporate EC in sport psychology practices. For instance, EC may be combined with behavioral interventions to improve coach–athlete relationships. On one hand, interpersonal behaviors are essential for good relationships. Coaches need to improve their explicit behaviors towards athletes; otherwise the positive effects of EC may be gradually cancelled out by negative interpersonal interactions in daily life. On the other hand, people in interpersonal relationships are also directed by implicit processes occurring automatically without awareness (McNulty and Olson, 2015; Hicks and McNulty, 2019). Improvement in behaviors alone do not necessarily lead to improvement in emotions and relationship satisfaction (Williamson et al., 2016). Therefore, interventions may be most effective if they target both explicit behaviors and implicit affective associations.

### Conclusion

The present study suggests that EC may be adopted as an effective intervention for coach–athlete relationships. It alters athletes’ affective associations with coaches to be more positive and improves their explicit evaluation of the relationships.

## Data Availability Statement

The raw data supporting the conclusions of this article will be made available by the authors, without undue reservation.

## Ethics Statement

The studies involving human participants were reviewed and approved by Beijing Sport University and Hangzhou Normal University. Written informed consent to participate in this study was provided by the participants’ legal guardian/next of kin.

## Author Contributions

JL, BC, and YZ contributed to the conceptualization and design of the study. BC collected the data. JL and BC analyzed the data and wrote the first draft of the manuscript. YZ reviewed and commented on the manuscript. All authors contributed to the article and approved the submitted version.

## Funding

This work was supported by the Starting Research Fund from Hangzhou Normal University under Grant No. RWSK20200407, the National Natural Science Foundation of China under Grant No. 32071087, and the Fundamental Research Funds for the Central Universities (Beijing Sport University) under Grant No. 2016QN017. The funders had no role in study design, data collection and analysis, decision to publish, or preparation of the manuscript.

## Conflict of Interest

The authors declare that the research was conducted in the absence of any commercial or financial relationships that could be construed as a potential conflict of interest.

## Publisher’s Note

All claims expressed in this article are solely those of the authors and do not necessarily represent those of their affiliated organizations, or those of the publisher, the editors and the reviewers. Any product that may be evaluated in this article, or claim that may be made by its manufacturer, is not guaranteed or endorsed by the publisher.
